# A commentary on the 2025 Annual Conference (Liverpool) Session ‘Beyond the lab – Turning your research into reality’

**DOI:** 10.1099/mic.0.001614

**Published:** 2025-10-14

**Authors:** Nicola Holden, Monika Gostic, Geertje van Keulen

**Affiliations:** 1 SRUC, School of Veterinary Medicine and Biosciences, Aberdeen, AB21 9YA, UK; 2 School of Medicine, Medical Sciences and Nutrition, The University of Aberdeen, Aberdeen, AB24 3FX, UK; 3 Swansea University, Medical School, Institute of Life Science, Swansea, SA2 8PP, UK

**Keywords:** business, impact, policy, translation of research

## Abstract

A session at the annual conference of the Microbiology Society 2025 (Liverpool) was held on gaining impact from basic and applied research, ‘Beyond the lab – Turning your research into reality’. This Commentary provides a short description of the rationale behind the session, the key take-home messages, information on the speakers and key resources they shared with the microbiology community.

## Introduction

There is much more to getting basic research into the clinic or the marketplace than just having a good idea and proving it works. Regulation and policy create the enabling environment within which research can be used in real-world settings and gain access to existing and new markets. Awareness of these powerful levers as key facilitators, and the constraints that may be placed upon a research outcome, should be included from the initial planning of the research to ensure success in translation and to avoid lengthy, time-consuming delays later. Similarly, most academic research outputs require transitioning into a commercially viable enterprise or industry-relevant tools and knowledge to achieve impact. Understanding how investors evaluate investment opportunities and the journey towards commercial success is also important.

A session at the annual Microbiology Society conference 2025, ‘Beyond the lab – Turning your research into reality’, included presentations from those involved in Policy, Regulation and investors, and examples from researchers to bring these topics to life for bench scientists. The session kicked off with Isabel Webb (UK Government, Department for Science, Innovation and Technology) [1], providing an update from the UK Government and explaining how policy meets progress. Matthew Gilmour (Quadram Institute) explained the importance of partnerships between industry and researchers, with real-world examples from two UK Research and Innovation (UKRI)-funded networks: Food Safety Research Network [2] and the AntiMicrobial Resistance in Agrifood Systems Transdisciplinary network (AMAST) [3]. We switched to examples for clinical and health-related research from Jo Fothergill (University of Liverpool), explaining the power of harnessing microbiomes [4]. Jonny Hazell (The Royal Society) explained the importance of underpinning evidence for regulatory policies [5] and the conundrums around using genetically modified micro-organisms for environmental benefit. Geertje van Keulen (Swansea University), one of our own chairs, described the newly formed Innovate UK-funded regulatory science network, Biofilm Alliance [6], which aims to bring together academic research, metrology and industry to advance regulatory guidance on biofilms through consensus. We then heard the exciting start-up journey taken by James MacDonald (Solena Materials Limited) for commercializing novel bio-based textile fibres, illustrating the importance of taking chance opportunities and the need to be truly interdisciplinary [7]. Daniel Robinson [8] described the investment UKRI is making to help cross the bridge between academic research and policy [9], reiterating the message regarding the importance of underpinning scientific evidence, no matter the discipline. The individual examples were rounded up by Sara Holland [10], explaining the value of intellectual property, what it means in reality and how to convert it into impact, while still meeting fundamental researcher goals [11]. A closing round-table discussion provided personal insight from the presenters on topical areas, balancing researchers’ needs for excellence with partner business or policy needs, and tips on how to gain traction with a research idea.

## Facing the uncertainty

For many (early-career) researchers, the idea of moving from bench to business sparks both excitement and deep uncertainty. For many academic researchers, the biggest barrier is not a lack of ambition or ideas; it is the insecurity, especially in not knowing how to get started. Yet, there are major benefits to taking this step, not just for the researcher involved but potentially much more widely. Impact can be felt in multiple ways. For example, a piece of evidence from one area may spark conversations in another. It may be highly relevant to grand challenges, like climate change or food security. Alternatively, combining multiple findings across disciplines could unlock the next step in an innovation pathway.

Key questions for overcoming the barriers are as follows:

- How do you move from a lab result or research paper to something investors, partners or policymakers care about?
- Where do you find the first opportunities to test your idea or build a prototype?
- Who teaches you how to pitch, not just your science, but its value and impact?

Impact does not happen by accident. It occurs when scientists are equipped not just with knowledge, but with guidance, mentorship and practical tools to navigate the transition. Enabling these discussions helps to normalize the fact that not knowing how to start is simply part of the process, and that there are multiple communities and routes to help. Science moves the world forward when the people behind it are supported and enabled to step beyond their comfort zones.

## Take-home messages

Collectively, the speakers raised key points on how to gain traction and impact from research activity. Their generously given top tips are encapsulated in five key take-home messages.

### All research activity and ideas are valuable

Everything that becomes applied or translated needs to have a solid foundation, completely reliant on a basic grounding in fundamental science and evidence. The success of those ideas and evidence can increase if they are rooted in solving a prioritized problem or meeting the need of key stakeholders. Databases exist that list strategic areas of interest [12], while contemporary challenges and cutting-edge applied science are featured in UK governmental [13] and parliamentary reports [14].

### Impact can be generated in a multitude of ways

Impact is not necessarily always measured by commercial output. It can also be relevant to policy or social enterprise. To understand the value of a microbiologist’s expertise and ideas, it is important to think about who is going to benefit, why and how, encapsulated in the three ‘Whats’: What is it, So What and What’s Next. This is exemplified by projects that have direct relevance to policy and regulation, e.g. in public health [2] or standards development [6], but equally for projects with potential commercial interest, whether from microbiome research [15] or from materials bioscience [7]. There may be regional considerations for where the impact will be felt and where it will have the most benefit. What may be key for developments to occur in a rural setting, for example, may not matter in urban environments. These questions are best answered by directly engaging with those who you think will benefit.

### Explore the different ways that the research can be used

While research provides data for a peer-reviewed paper, its utility does not need to stop there. One of the first steps is to understand the routes and options. Research organizations have innovation or enterprise centres with dedicated staff to offer advice. They can signpost researchers to professional services and training, like the UKRI-supported scheme ‘Fast Track Impact’ [16], to build necessary skills and confidence. University mentoring schemes provide innovative researchers access to those with experience for guidance and discussion of ideas. The Microbiology Society has relevant resources [17], while networks and forums have published further information and can be contacted for questions to help develop your ideas and to gain traction.

### Do not navigate the maze alone

There is no doubt that the transition from the lab towards application will move the typical lab microbiologist out of their comfort zone. All of the different component parts can seem like a bit of a maze to navigate, with communication and collaboration vital to help along the journey. Another tip was the benefits of using toolkits. These help to develop frameworks [18,19], providing much-needed structure for a stepwise progression through the maze.

### Look at funding options as a portfolio

Funding ideas is a fundamental requirement, and there are a number of options suited for different aspects. Funders like Innovate UK offer guidance for their different schemes [20], which may prioritize types of sectors or be suited to different stages of technology readiness levels, from feasibility testing to innovation loans. Other funding opportunities may be aligned with specific topics, be it biofilms [6,21] or microbiomes [15]. Alternatively, they may combine geographical regions with particular topics, like biotechnology [22]. Considering them as a basket or portfolio of options rather than a single one shows how different parts of the idea can be supported at different stages of development.

In all, the broad scope of the speakers’ backgrounds, their expertise and in-depth knowledge was invaluable to demystify the options, different stages and reasons for reaching beyond the lab. The next step may be a simple, initial discussion over a conference coffee break, reaching out to the local research innovation centre or signing up for an online course; the key is taking that step with the full knowledge that resources are there to help.

## Resources from the session

### Speakers and presentation titles

Isabel Webb (UK Government, Department for Science, Innovation and Technology) ‘Update from the Department for Science, Innovation and Technology’

Matthew Gilmour (Quadram Institute) ‘Building partnerships between industry and researchers to tackle the ‘problems worth solving by us’

Jo Fothergill (University of Liverpool) ‘Harnessing the Microbiome: From Academic Insight to Impact’

Jonny Hazell (The Royal Society) ‘Regulating genetically modified microorganisms for environmental benefit’

Geertje van Keulen (Swansea University) ‘Biofilm Alliance: A Network for Regulatory Sciences, Academic Research, and Industry Collaboration’

James MacDonald (Solena Materials Limited) ‘From Chance Meeting to Startup – The Journey of Commercialising Novel Biobased Textile Fibres’

Daniel Robinson (Economic and Social Research Council) ‘How and Why to get Involved in Policy’

Sara Holland (Potter Clarkson) ‘Research to real impact through IP’

## Web links

### Societies

- Microbiology Society policy
- Royal Society GMO and regulations

### UK Government, agencies

- DSTL
- GO Science
- POST notes
- Areas of Research Interest database
- UKRI policy fellowships
- Capability in Policy Engagement

### Training & skills

- Fast Track Impact
- Nesta toolkit for public innovation
- IP FAQs for start-ups (Potter Clarkson)

### Academic-industry networks

- FSRN
- UKRI funded networks on AMR
- Biofilm Alliance

## Abbreviations

UKRI: UK Research and Innovation councils

## References

[1] DSIT (2025). https://www.gov.uk/government/organisations/department-for-science-innovation-and-technology.

[2] Food safety research network (2025). Home. https://fsrn.quadram.ac.uk/.

[3] AMAST Network (2025). About. https://amast.org.uk/.

[4] University of Liverpool (2025). Microbiome innovation centre. https://www.liverpool.ac.uk/microbiome-innovation-centre.

[5] Royal Society (2025). Evidence-led GM crop regulation could help UK lead on tackling global food security and climate change risks, says Royal Society. https://royalsociety.org/news/2023/10/gm-crops/.

[6] Biofilm Alliance (2025). Home. https://www.biofilm-alliance.org/.

[7] Solena Materials (2025). Solena. https://www.solena-materials.com/.

[8] UKRI (2025). Economic and Social Research Council (ESRC). https://www.ukri.org/councils/esrc/.

[9] UKRI (2025). https://www.ukri.org/opportunity/ukri-policy-fellowships-2025/.

[10] Potter Clarkson (2025). https://www.potterclarkson.com/.

[11] Potter Clarkson (2025). What are the key IP considerations for technology start-ups?. https://www.potterclarkson.com/insights/what-are-the-key-ip-considerations-for-technology-start-ups-part-1/.

[12] ARI (Areas of Research Interest) (2025). Where can research make a difference?. https://ari.org.uk/.

[13] UK Government (2025). Government office for science. https://www.gov.uk/government/organisations/government-office-for-science.

[14] UK Parliament (2025). POSTnotes. https://post.parliament.uk/type/postnote/.

[15] UKRI (2025). Microbiome. https://iuk-business-connect.org.uk/agrifood/microbiome/.

[16] Fast Track Impact (2025). About. https://www.fasttrackimpact.com/for-researchers.

[17] Microbiology Society (2025). Why microbiology matters. https://microbiologysociety.org/why-microbiology-matters.html.

[18] CAPE (2025). The art of the possible: catalysts, collaborations and capabilities in academic-policy engagement. https://www.cape.ac.uk/resources/.

[19] Nesta (2025). Skills, attitudes and behaviours that fuel public innovation. https://www.nesta.org.uk/toolkit/skills-attitudes-and-behaviours-fuel-public-innovation/.

[20] UKRI (2025). Innovate UK: guidance for applying to specific funds. https://www.ukri.org/councils/innovate-uk/guidance-for-applicants/guidance-for-specific-funds/.

[21] National Biofilms Innovation Centre (2025). Opportunities at NBIC. https://biofilms.ac.uk/nbic-funding-opportunities/.

[22] IBioIC (2025). Funding opportunities from IBioIC. https://www.ibioic.com/funding-opportunities-from-ibioic.

